# Chemical glycomics enrichment: imaging the recycling of sialic acid in living cells

**DOI:** 10.1007/s10545-017-0118-3

**Published:** 2018-01-02

**Authors:** Pierre André Gilormini, Cédric Lion, Dorothée Vicogne, Yann Guérardel, François Foulquier, Christophe Biot

**Affiliations:** University Lille, CNRS, UMR 8576 - UGSF - Unité de Glycobiologie Structurale et Fonctionnelle, F-59000 Lille, France

## Abstract

The development of metabolic oligosaccharide engineering (MOE) over the past two decades enabled the bioimaging studies of glycosylation processes in physio-pathological contexts. Herein, we successfully applied the chemical reporter strategy to image the fate of sialylated glycoconjugates in healthy and sialin-deficient patient fibroblasts. This chemical glycomics enrichment is a powerful tool for tracking sialylated glycoconjugates and probing lysosomal recycling capacities. Thus, such strategies appear fundamental for the characterization of lysosomal storage diseases.

## Introduction

Sialic acid storage disease (SSD) is an autosomal recessive lysosomal storage disorder that mainly affects the patient’s nervous system. This condition is generally classified into one of three forms, namely a severe infantile SSD form (ISSD), a milder adult form originally reported in patients from Salla in Finland (Salla disease, SD) or an intermediate severe Salla disease (Aula et al 1979). Recently, a literature review reported 194 patients with SD (Barmherzig et al 2017). The main symptoms comprise intellectual disability and global developmental delay, hypotonia, seizures, ataxia, and muscle spasticity. While patients with SD usually survive into adulthood, children affected with the ISSD form display more severe symptoms, such as failure to thrive, enlarged organs or abnormal fluid build-up, and usually live only into early childhood. Salla disease is caused by mutations (including R39C and K136E) in the *SLC17A5* gene encoding the transporter protein sialin of 495 amino acids (Morin et al 2004; Miyaji et al 2011). SLC17 is a functionally diverse family of organic anion transporters composed of nine members distributed into two subfamilies: the Na^+^-phosphate co-transporters and the vesicular transporters. The vesicular transporter subfamily includes five members: the vesicular excitatory amino acid transporters (SLC17A5), the vesicular glutamate transporters (SLC17A6, SLC17A7, and SLC17A8), and the vesicular nucleotide transporters (SLC17A9) (Miyaji et al 2008; Togawa et al 2015). The SLC17A5 protein or sialin is found in lysosomes where it plays a role in the export of *N*-acetyl-D-neuraminic acid (Neu5Ac) (Pietrancosta et al 2012). In human cells, Neu5Ac is by far the most prominent member of the sialic acid family, a group of nine-carbon sugar acids found predominantly at the termini of cell-surface glycans that are recognized by endogenous and exogenous receptors and have essential functions in both physiological and pathological contexts. The initial steps in biochemical diagnosis are urinalysis of Neu5Ac and also its detection in tissue samples and cultured fibroblasts.

## Metabolic labeling of glycans

**Fig. 1:**
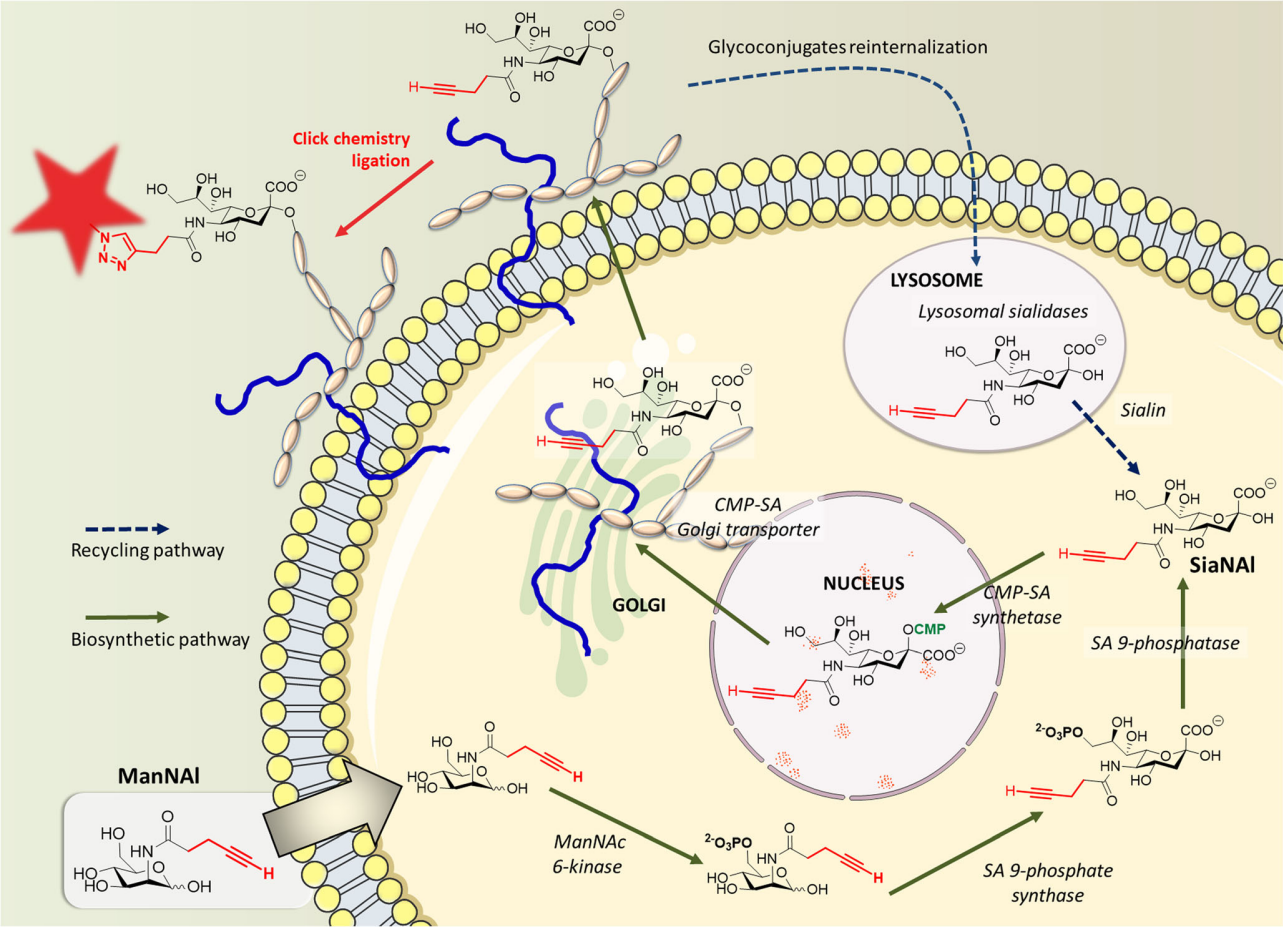
Schematic representation of the biosynthetic (green arrow) and the recycling (blue dashed arrow) pathways of sialic acids into mammalian cells. The alkynyl reporter Man*N*Al enters the cell and is enzymatically modified into Sia*N*Al in the cytosol. Then, its activation is performed in the nucleus before transport into the Golgi and incorporation into the glycans through the action of sialyltransferases. The incorporated alkyne can then be detected via chemical ligation with a specific fluorescent probe. The reinternalization of the glycoconjugates is achieved by endocytosis. After maturation of the endosomes into lysosomes, specific lysosomal sialidases are able to cleave the terminal sialic acids residues which are subsequently transported from the lysosome to the cytosol by the specific transporter sialin

In the early 1990s, Werner Reutter’s group treated rats with *N*-acetyl-D-mannosamine analogues in which the acetyl group was replaced by a propanoyl group (Man*N*Prop) (Kayser et al 1992). Their results showed that not only was Man*N*Prop successfully taken up by the cells, but also that it was efficiently converted into the corresponding *N*-acetyl-neuraminic acid analogue Neu5Prop and subsequently incorporated into glycoconjugates. This pioneering work suggested that, owing to the promiscuous nature of the enzymes of the Roseman-Warren pathway that describes the de novo biosynthesis of Neu5Ac, mannosamine analogues with a modified *N*-acyl side-chain could be converted to the respective unnatural sialic acid by the metabolic machinery of the cell and be expressed at the cell surface. Inspired by this first example of metabolic oligosaccharide engineering (MOE), Carolyn R. Bertozzi’s group improved the strategy in 1997, giving birth to the chemical reporter strategy that enables detection of the modified sialoglycans (Mahal et al 1997). This methodology is divided into two well-defined steps: (i) a synthetically modified monosaccharide bearing a chemical handle (the reporter) that is both non-reactive toward living systems and absent of cells, is first introduced into an organism (cells, living animal…). Hijacking the natural biosynthetic pathways, the modified monosaccharide is incorporated into nascent glycoconjugates and the reporter is exhibited at the surface of cells. (ii) the introduced reporter is then reacted with a complementary bioorthogonal chemical function, which itself is linked to a probe (e.g., biotin, fluorescent dyes, crosslinking reagents) thereby allowing the specific detection/imaging of the modified glycoconjugate (Fig. 1). Note that the unnatural monosaccharide must contain a modification that does not disturb its recognition by enzymes and incorporation into glyconjugates. Pioneered by the Bertozzi laboratory, several chemical groups and bioorthogonal reactions have been developed in order to achieve the probe/reporter ligation in a specific manner. MOE has been applied widely in the last two decades, in mammalian cells (Mahal et al 1997; Vocadlo et al 2003; Hang et al 2003; Sampathkumar et al 2006; Hsu et al 2007; Laughlin et al 2008; Cole et al 2013; Stairs et al 2013; Chuh et al 2014; Späte et al 2014; Rodriguez-Rivera et al 2017), in bacteria, (Dumont et al 2012; Fugier et al 2015), in plants (Anderson et al 2012; Dumont et al 2016; Sminia et al 2016) and into organs (Jiang et al 2014) or even into animals (Laughlin and Bertozzi 2009; Jiang et al 2014; Xie et al 2016). In addition, MOE has been used to achieve proteomic analysis of different glycoproteins (Woo et al 2015; Sun et al 2016). However, among the applications of MOE, there are only a very few reports describing the subcellular visualization of glycoconjugate trafficking, either in physiological conditions or in pathological conditions such as glycosylation defects. In 2013, Mbua and co-workers applied MOE to the visualization of glycoproteins which are accumulated into lysosomes of Niemann-Pick type C disease patients (Mbua et al 2013). Later that same year, we reported the use of MOE as an evaluation tool for congenital disorders of glycosylation. Indeed, we showed that Golgi-staining intensity after feeding with our modified monosaccharide varied depending on the pathology of the cell (Vanbeselaere et al 2013). More recently, we developed an original sequential bioorthogonal dual strategy (SBDS) in which we examined differences of uptake between two non-peracetylated alkyne analogues of Man*N*Ac and Neu5Ac, respectively, Man*N*Al and Sia*N*Al (Gilormini et al 2016). Our strategy allowed us to clearly visualize a sialin deficiency in patient cells and to get an insight into the uptake mechanisms of sialic acids and their precursors. Indeed, our results strongly reinforced the hypothesis of Varki's group stating that exogenous sialic acid enters the cell via endocytosis (Bardor et al 2005). Furthermore, we suggested that the entry of Man*N*Ac analogues through the plasma membrane is mediated by a yet unknown specific transporter.

After investigating the uptake processes of Man*N*Ac analogues, we present here our application of MOE to get an insight into the fate of the sialylated glycoconjugates. Although the recycling processes of glycoconjugates are not well known, some interesting data were reported by Reutter and co-workers in the 1980s. The half-life times of different monosaccharides were studied. For example, terminal monosaccharides, such as fucose and sialic acids, showed fast half-life rates (12.5 h for L-fucose, 30 h for Neu5Ac) compared to residues from the *N*-glycan core (D-mannose, 70 to 130 h) or to the protein moiety (100–130 h) (Kreisel et al 1980; Tauber et al 1983). From these observations, the researchers were able to show that a single protein could go through several cycles of sialylation during its lifetime. Membrane glycoproteins are therefore supposed to be able to be reinternalized by endocytosis, and deglycosylated partially into lysosomes. These proteins subsequently join back with the secretion pathway in the Golgi apparatus where they can be glycosylated again (Kreisel et al 1988). New tools and methodologies for the study and evaluation of recycling processes and turnover rates of the glycoconjugates are of great interest for a better understanding of these under-reported processes. Indeed, it has been shown that turnover rates of glycans chains were faster in tumoral cells compared to healthy cells (Tauber et al 1989). Therefore, an extensive study of these turnover mechanisms could provide new insights into some pathologies and/or infections involving the glycans. In the present work, we have applied MOE principles to compare the *in cellulo* visualization of sialylated glycoconjugates between wild-type and sialin deficient fibroblasts.

## Materials and methods

### General methods

Chemicals reagents were purchased from Sigma Aldrich, TCI and Carbosynth and were used with no further purification. Anti LAMP2 was from Santa Cruz Biotechnology (Santa Cruz, CA, USA).

### Synthetic procedures

*N*-(4-pentynoyl) mannosamine (Man*N*Al) was synthesized according to optimized procedures (Gilormini et al 2016). The dynamics of incorporation into glycoconjugates and their localization was monitored by chemical ligation of commercially available azido-functionalized fluorophores. We used the biocompatible ligand-mediated copper-catalyzed azide-alkyne [3 + 2] cycloaddition (CuAAC) before imaging by confocal fluorescence microscopy (Gilormini et al 2016). BTTAA was synthesized as previously described (Besanceney-Webler et al 2011; Yang et al 2014).

### Cell culture

Primary skin fibroblasts (MW28 and sialin deficient patient cells (SLC17A5, 1-BP DEL, 533C), kindly provided by Dr. Thierry Levade, were maintained in Dulbecco’s modified Eagle’s medium (DMEM) supplemented with 10% fetal bovine serum (Lonza) at 37 °C in humidity saturated 5% CO_2_ atmosphere. When used, chloroquine (CQ) was added to the culture medium at the final concentration of 40 μM.

### Metabolic labeling with alkyne tagged analogs

Fibroblasts were grown overnight on glass coverslips (12 mm diameter). Medium was then changed with pre-warmed medium containing 500 μM of ManNAl. The labeling was stopped at the different time points mentioned by fixing the cells with 4% paraformaldehyde (PAF). Cells were then permeabilized in 0.5% Triton X-100 for 10 min. Permeabilized cells were then incubated with 100 μL/coverslip of a freshly prepared click solution (K_2_HPO_4_, 100 mM; Sodium ascorbate, 2.5 mM; CuSO_4_, 150 μM; BTTAA, 300 μM, AzidoFluor 545, 10 μM). CuAAC was performed during 45 min, in the dark, at room temperature with gentle shaking. After 2 h saturation in blocking buffer (0.2% gelatin, 1% BSA and 2% normal goat serum (Invitrogen) in PBS), fixed cells were incubated at room temperature for 1 h with Alexa 488-, Alexa 568-, and Alexa 700-conjugated secondary antibodies (Molecular Probes) diluted at 1/600 in blocking buffer.

### Imaging

Immunostaining and fluorescent proteins were detected through an inverted Leica TCS-SP_5_ confocal microscope. Pictures were taken by using Leica Application suite Advanced Fluorescence (LAS AF) software (Leica Microsystems Wetzlar, Germany). For comparison purposes, each picture was taken under the same settings. For quantification, we used the Leica TCS-SP_5_ intensity plotting tool that provides relative fluorescence intensities in different collection channels over a region of interest (ROI). A plot of fluorescence intensity in a ROI corresponding to the Golgi region was performed for each cell. For image analysis, three different fields of two independent experiments were examined. Around 100 cells were quantified. The LAS AF pictures were then exported in TIFF format and processed with Adobe Photoshop 7.0.

### Sialidase treatment

Control fibroblasts were grown overnight on glass coverslips (12 mm diameter). Medium was then changed with pre-warmed medium containing 500 μM of Sia*N*Al overnight. After fixation with paraformaldehyde 4%, cells were treated to recombinant sialidase C from *C. perfringens* (Prozyme # GK80030) in PBS for 1 h at 37 °C. Cells were subsequently washed with PBS, permeabilized with Triton X-100 0.5% for 10 min, and finally stained following our CuAAC procedure previously described.

### Statistics

None of the experiments were blinded and no statistical methods were used to pre-determine sample size for in vitro experiments.

## Results and discussion

### Pulse chase experiment

**Fig. 2:**
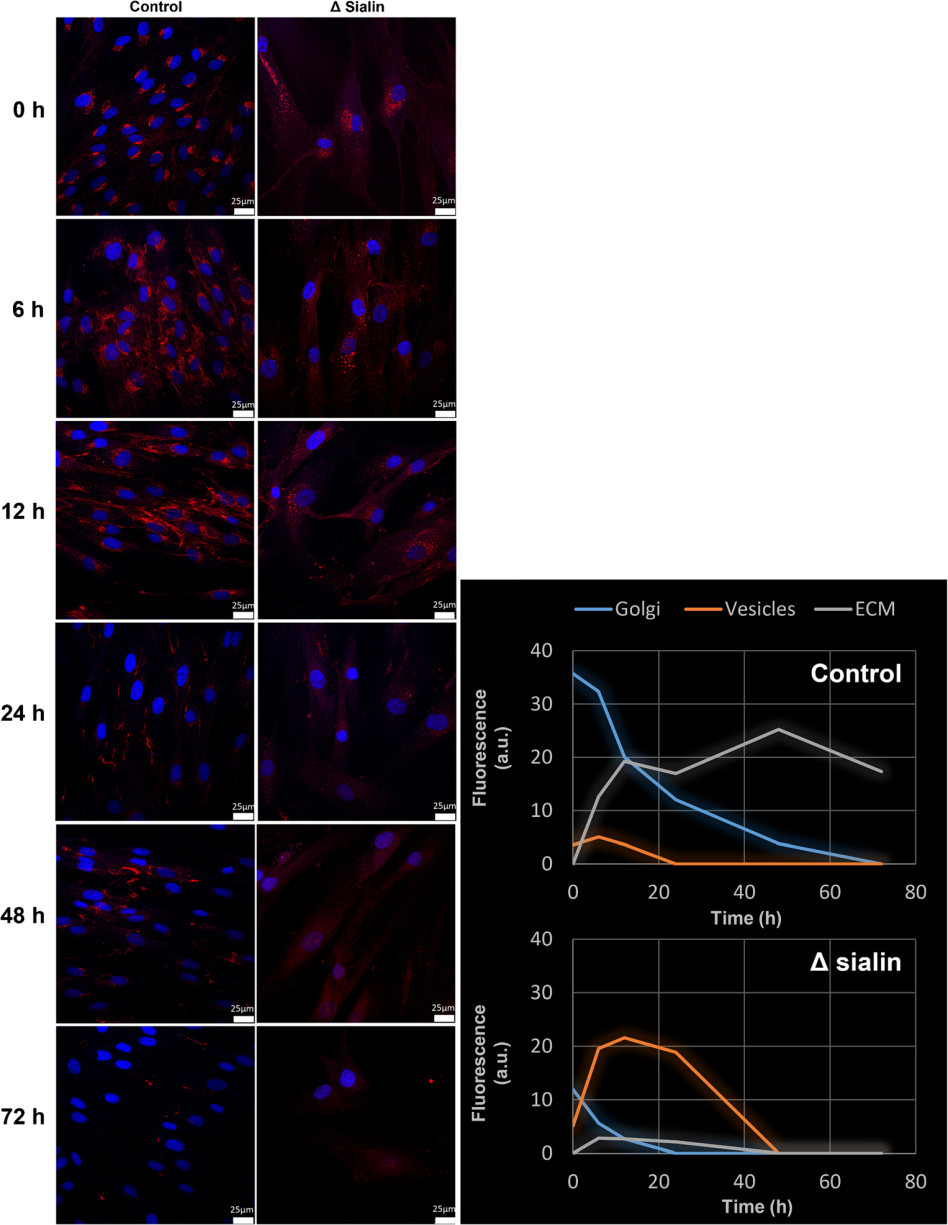
Fibroblasts from healthy individuals and sialin deficient patient were metabolically labeled with 500 μM of Man*N*Al for 8 h (T_0_) and then chased for 6, 12, 24, 48 and 72 h. The sialylated glycoconjugates (in red) were visualized by confocal microscopy after staining with azido 545 fluorescent probe (Scale bar, 25 μm)

**Fig. 3:**
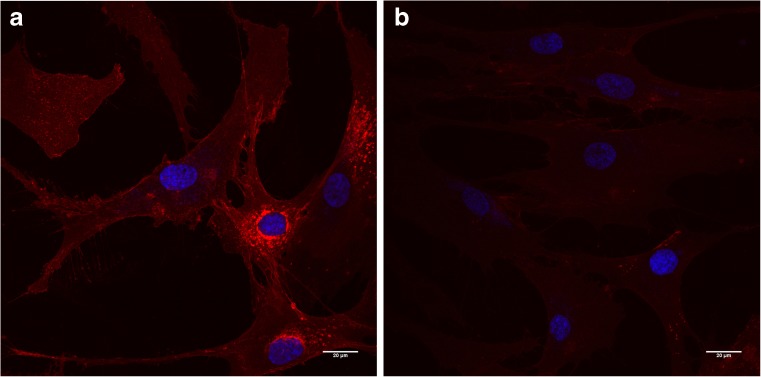
Fibroblasts from healthy individual were metabolically labeled with 500 μM of Sia*N*Al overnight and then fixed and submitted to CuAAC reaction with Azido 545 fluorescent probe (red signal) and DAPI (blue signal). Immediately after fixation, cells were submitted to either no treatment (**a**) or *Arthrobacter ureafaciens* sialidase treatment (**b**) (Scale bar, 20 μm)

In order to investigate the dynamics of sialylated glycoconjugate recycling, we applied our metabolic labeling strategy to pulse−chase experiments using Man*N*Al as the reporter. Fibroblasts from both healthy and sialin deficient patient were cultured in the presence of 500 μM of our alkyne tagged sugar Man*N*Al in DMEM for 8 h (pulse). Man*N*Al containing medium was then replaced by DMEM and the fibroblasts were grown for up to 72 h (chase). After 0, 6, 12, 24, 48 or 72 h, cells were fixed, permeabilized, and reacted with the fluorescent probe azidoFluor 545 in the presence of CuSO_4_ (150 μM) and of the tris(triazolylmethyl)amine ligand BTTAA (300 μM). All the conditions were imaged by confocal microscopy and are presented in Fig. 2. At T_0_, meaning after 8 h of incubation with Man*N*Al, both healthy and sialin deficient fibroblasts show a red stained Golgi-like perinuclear area. After 6 h of chase, differences are observed between the two cell lines. In control cells, Golgi staining decreases slowly and a clear labeling of the plasma membrane can be observed after 12 h of chase. As glycoconjugates are known to be sialylated in the Golgi and subsequently addressed to the plasma membrane, our observations between T_0_ and T_12_ are consistent with the literature. At T_24_, there is a strong decrease of the total signal which significantly reappears at T_48_ and finally vanishes at T_72_. These data strongly suggest that the alkyne-tagged neo-sialylated glycoconjugates are reinternalized by endocytosis and submitted to enzymatic cleavage of terminal sialic acids in the lysosomes. Indeed, it is important to stress that cell samples are permeabilized and washed several times after staining procedures: as a consequence, free soluble alkynyl monosaccharides are washed away and thereby not detected during confocal microscopy image acquisition. The signal observed in these experiments exclusively corresponds to poly- or oligosaccharidic glycoconjugates that are metabolically labeled with the alkyne reporter. The reappearance of the fluorescence signal between T_24_ and T_48_ therefore strongly suggests that our alkyne reporter is re-introduced into the metabolic pathway (after degradation of the glycoconjugate it was first incorporated into) and recycled once again in the glycoconjugate metabolism, explaining the increase of the signal at T_48_. After 72 h, no staining can be observed anymore, probably because of the catabolism of the alkyne monosaccharide combined to signal dilution due to cellular growth. This is to our knowledge the first time that the turnover of sialoconjugates is imaged by MOE strategy. At this point, confirmation was needed to evaluate the potential non-recognition of our alkynyl analog by sialidase(s). Indeed, there is evidence that the chemical modification of sialic acids on the C5 position affects their recognition and releasing by bacterial sialidases (Cao et al 2009; Heise et al 2017). We thus incubated control fibroblasts with 500 μM of Sia*N*Al overnight. Then cells were fixed and treated with *Arthrobacter ureafaciens* sialidase before our staining procedure with CuAAC. The use of the sialidase induced an almost complete disappearance of the signal compared to the same treatment without sialidase (Fig. 3). This result confirms the tolerance of sialidase for our alkyne reporter and then enforces our hypothesis concerning the visualization of sialic acid turnover.

Sialin deficient cells present a completely different pattern. The function of sialin is to transport free sialic acid out of the lysosome after it is cleaved from sialylated glyconconjugates undergoing degradation. The total lack of signal at T_48_ and T_72_ therefore reflects the absence of sialin: the lack of this lysosomal transporter prevents the export of free sialic acid from lysosomes to the cytosol and thus its recycling into “second generation” neoglycoconjugates. However, the localization of glycoconjugates in sialin deficient cells between T_0_ and T_12_ raises some interrogations. While the vesicular punctuated staining was barely visible in control cells, it was obvious in sialin deficient cells after 6 h of metabolic incorporation suggesting that sialoglycoonjugates never reached the cellular membrane but transited through vesicles.

### Colocalization with Lamp-2

**Fig. 4:**
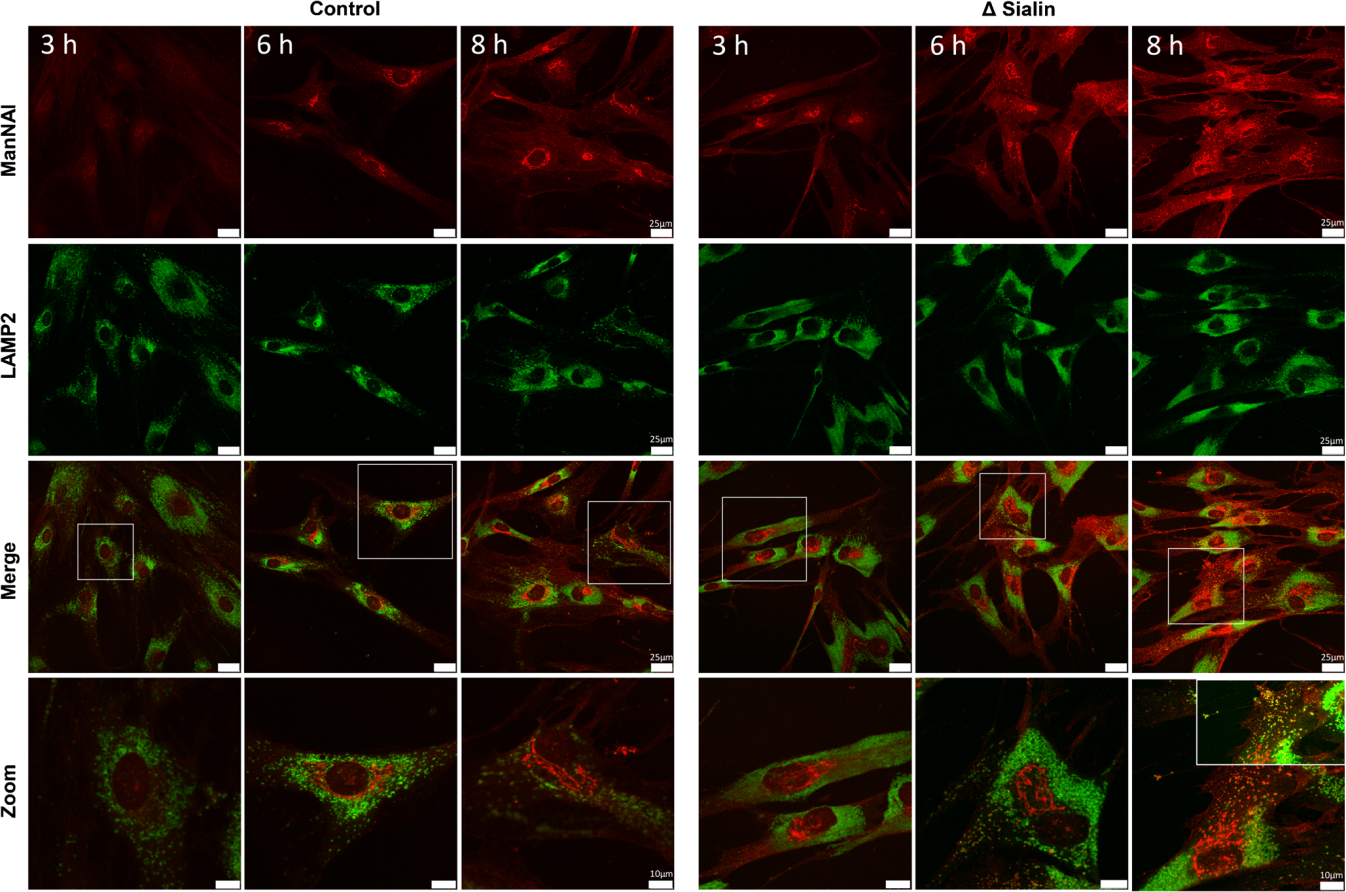
Fibroblasts from healthy individuals and sialin deficient patient were metabolically labeled with 500 μM of ManNAl for 3, 6, and 8 h respectively and stained with azido 545 fluorescent probe (sialic acid in glycoconjugates in red) or antibodies against a lysosomal marker (LAMP-2 in green). Staining was then visualized using confocal microscopy. It could be noticed that this co-localization of the MOE signal with the lysosomal LAMP-2 marker was seen in all investigated cells (Scale bar, 25 μm)

Suspecting that this vesicular staining co-localized with lysosomes, we repeated the MOE experiment with Man*N*Al using LAMP-2 as a lysosomal membrane marker (Fig. 4). While no co-localization could be identified between LAMP-2 and our reporter in control cells, we observed a co-staining in sialin deficient fibroblasts. Since no labeling would be observed if the sialic acid residues were cleaved and released as free monosaccharides owing to the washing protocol, we thus conjectured that this lysosomal fluorescence signal reflected the accumulation of sialylated glycoconjugates in the lysosomes. This accumulation could be due to a dysfunction of the lysosomal sialidases, enzymes that hydrolyze the glycosidic linkages of the terminal sialic acid residues of glycoconjugates. The optimal pH for lysosomal sialidase activity has been reported between 4.2 and 4.6 (Thomas et al 1979). Under physiological conditions, Neu5Ac is present as the negatively charged carboxylate conjugate base form. This led us to the hypothesis that the accumulation of free sialic acid in the lysosomes could have an influence on the lysosomal pH thereby hindering sialidase activity.

### Chloroquine treatment

**Fig. 5:**
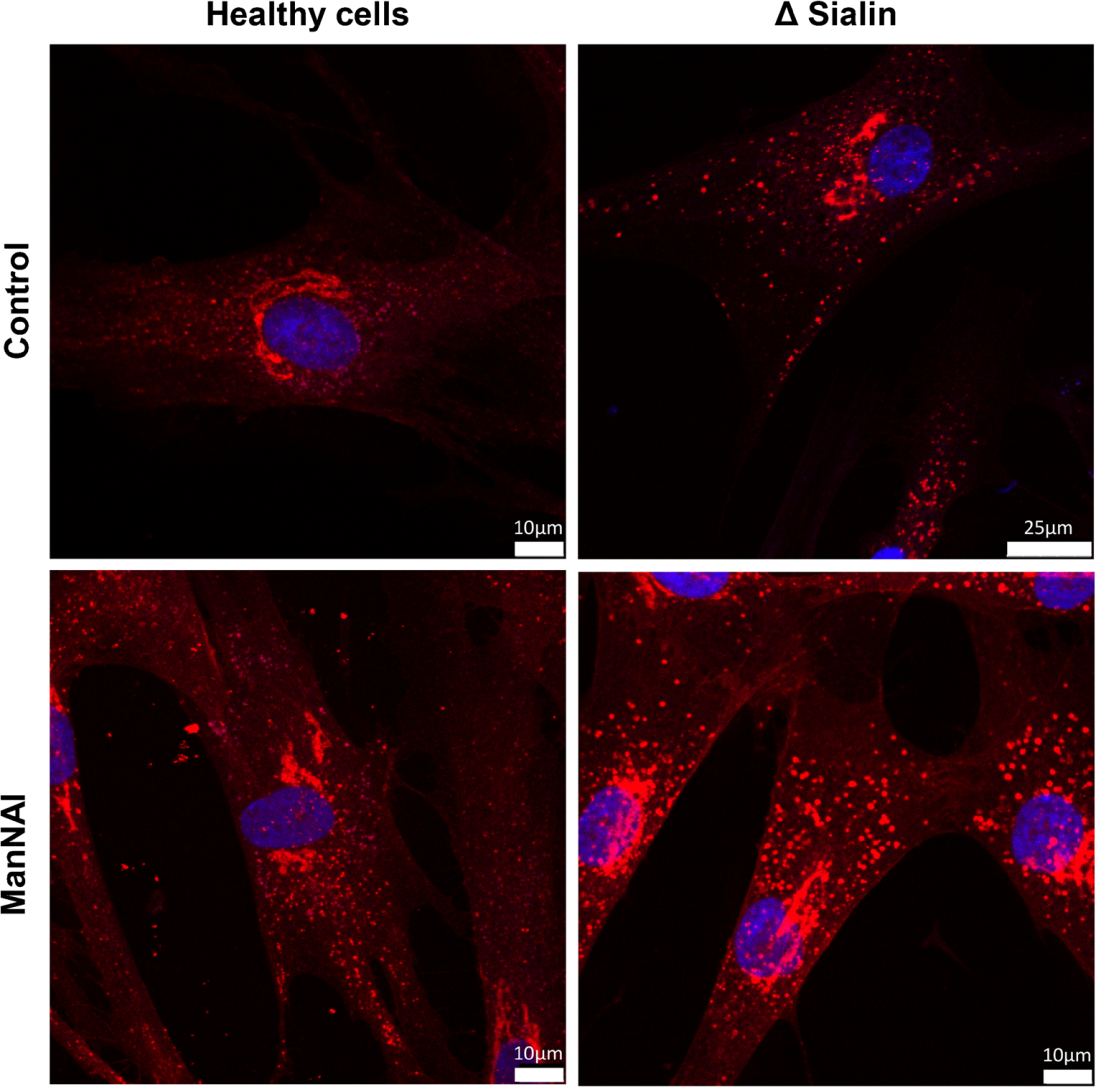
Fibroblasts from healthy individuals and sialin deficient patients were metabolically labeled with 500 μM of Man*N*Al for 8 h in absence or presence of 40 μM chloroquine and stained with azido 545 fluorescent probes (sialic acid in glycoconjugates in red). Staining was visualized using confocal microscopy (Scale bar, 10 μm except for image on the up-right, 25 μm)

To prove our hypothesis, we decided to artificially increase the lysosomal pH of fibroblasts. To this end, we used the antimalarial drug chloroquine (CQ), a diprotic weak base (pKa 8.4 and 10.8) that is also known as a lysosomotropic agent, preferentially accumulating in lysosomes by pH trapping. Fibroblast cells were treated with 40 μM chloroquine for 16 h in order to inhibit lysosomal degradation before incubation with Man*N*Al. After the usual staining process, cells were observed by confocal microscopy (Fig. 5). A clear increase of lysosomal staining intensity was observed in both control and sialin deficient fibroblasts while no changes were noted in the Golgi apparatus. These data show that, while the sialic acid biosynthetic pathway remains unchanged, the pH increase induced by CQ clearly leads to an accumulation of sialylated glycoconjugates in the lysosomes, with or without sialin activity.

## Conclusion

In conclusion, we proposed here a MOE procedure, coupled with biorthogonal ligation for the visualization of the recycling and turnover of sialic acids. The use of sialin-deficient cells allowed us to image the glycoconjugate recycling pathway. The chemical reporter strategy, applied to the visualization of intracellular processes and combined to traditional methodologies, clearly has the potential to provide exciting insights into glycosylation mechanisms.

## Abbreviations

BTTAA: 2-(4-((bis((1-tert-butyl-1H-1,2,3-triazol-4-yl)methyl)amino)methyl)-1H-1,2,3-triazol-1-yl)acetic acid
CRS: Chemical reporter strategy
DMEM: Dulbecco’s modified Eagle’s medium
ISSD: Infantile sialic acid storage disease
Man*N*Ac: *N*-acetyl-D-mannosamine
Neu5Ac: *N*-D-acetylneuraminic acid
SD: Salla disease
SASDS: Sialic acid storage diseases
